# The Association Between Race and Complications in Free Flap Surgery of the Head and Neck

**DOI:** 10.7759/cureus.104557

**Published:** 2026-03-02

**Authors:** Andrea Jakubowski, Jeremy Walsh, Ryan Nagy, David Riccio, Adrian A Ong, Gaayathri Varavenkataraman, Michele M Carr

**Affiliations:** 1 Otolaryngology, Lake Erie College of Osteopathic Medicine, Erie, USA; 2 Surgery, Jacobs School of Medicine and Biomedical Sciences, University at Buffalo, Buffalo, USA; 3 Emergency Medicine, Memorial Health Systems, Marietta, USA; 4 Otolaryngology, Jacobs School of Medicine and Biomedical Sciences, University at Buffalo, Buffalo, USA

**Keywords:** free flap reconstruction, head and neck cancer, national surgical quality improvement program, nsqip database, postoperative complications, racial disparities, surgical complications

## Abstract

Objective

The objective of this study was to assess the impact of race on outcomes in patients undergoing free flap reconstruction of the head and neck.

Methods

Data were taken from the National Surgical Quality Improvement Program (NSQIP) database for surgical procedures relating to free flap reconstruction of the head and neck using appropriate Current Procedural Terminology (CPT) codes from 2017 to 2019. Outcomes were initially compared by race. A sub-analysis was performed after propensity score matching between those who identified as Caucasian (White) and Black or African American in race.

Results

In total, 1589 patients underwent head and neck free flap surgeries between 2017 and 2019. Of those, 1075 patients identified as Caucasian and 176 identified as non-Caucasian, with 94 identifying as Black or African American. Prior to propensity score matching, Caucasian patients were significantly older, more likely to have moderate dyspnea, more likely to be partially functionally dependent, and less likely to require a ventilator preoperatively than non-Caucasian patients. There were no significant differences in length of stay, readmission, and reoperation between patients identifying as Caucasian and patients identifying as Black or African American.

Conclusion

For patients included in the NSQIP database, race does not appear to impact outcomes in those undergoing free flap reconstruction of the head and neck.

## Introduction

Head and neck cancer is a significant cause of morbidity and mortality, ranking as the seventh most common type of cancer worldwide. In 2016, 4.1 million cases of head and neck cancer were reported globally, resulting in approximately 500,000 deaths [1]. Over the last decade, the incidence of oral cavity and pharyngeal cancer has increased by 0.6% per year [2]. This anatomical region is complex, with numerous functions related to both quality of life and survival. Cancers vary significantly in the region, affecting a wide variety of cell types and anatomical structures. Numerous risk factors for head and neck cancer have been identified, including alcohol and tobacco use, betel nut use, poor dental hygiene, and transmission of human papillomavirus (HPV) and Epstein-Barr virus (EBV) [2]. Treatment options may include surgery, radiation therapy, immunotherapy, chemotherapy, or combination therapy, depending on several factors.

Free tissue transfer for surgical reconstruction has become a mainstay in patients with head and neck cancer. Though free tissue transfer has revolutionized the management of head and neck cancer, concerns arise regarding complications affecting vital functionality and aesthetic outcomes. While free flaps often have a greater than 95% success rate, perioperative optimization helps to reduce complications and free flap failure, reducing the need for readmission and reoperation [3]. Predicting complications and surgical planning for high-risk groups can help optimize care and improve outcomes. Although there is variability between studies and confounding effects making it difficult to generate an ideal approach to these surgeries, many of these conditions are modifiable and can be optimized prior to surgery to improve surgical outcomes. For example, patients with head and neck cancer are often malnourished due to difficulty with swallowing, leading to more unfavorable outcomes following free flap reconstruction [4]. Other factors that may influence outcomes are non-modifiable, though determining those patients at risk can facilitate more focused care related to specific perioperative outcomes and complications. 

Previous studies have demonstrated inconsistent significance of race on postoperative outcomes and complications [5-7]. An increase in complications and more unfavorable outcomes has been shown in non-Caucasian races, specifically Black or African American patients. In bariatric, orthopedic, and total thyroidectomy surgeries, Black and non-Caucasian races were shown to be associated with an increased likelihood of readmission and postoperative complications [5-7]. Conversely, race has also been shown to have no independent significance on urologic cancer surgery outcomes, with comorbidity burden being the confounding factor [8]. To our knowledge, few studies have thus far examined the effect of race on postoperative outcomes and complications following head and neck free flap reconstruction surgeries. Cotton et al. assessed risk factors associated with the need for perioperative red blood cell transfusions (RBCT) in head and neck cancer patients undergoing free flap reconstruction, demonstrating African American race as a risk factor for RBCT [9]. Bhethanabotla et al. investigated the role of social determinants of health on clinical outcomes for patients undergoing osteocutaneous free flap reconstruction for mandibular defects due to oral cavity malignancy, ultimately finding no significant differences in clinical outcomes based on race. However, there exist numerous limitations to this study, which include a relatively small sample size, a lack of assessment of individual racial and ethnic subgroups, and a single institution focus [10]. Our study aims to aid the current literature in examining the role of demographics and perioperative characteristics in head and neck free flap surgical outcomes.

## Materials and methods

Study design

This was a retrospective analysis conducted using data from the American College of Surgeons National Surgical Quality Improvement Program® (ACS NSQIP®) database from January 1, 2017, to December 31, 2019. The NSQIP database is multi-institutional and collects information including demographics, comorbidities, perioperative factors, and 30-day morbidity and mortality outcomes. The data are collected by trained professionals and randomly audited for quality assurance. Cases in the NSQIP database were identified using Current Procedural Terminology (CPT) codes, and relevant data were extracted for analysis. Primary outcomes included length of stay, readmission, and reoperation. Secondary outcomes included postoperative complications. The study was classified as non-human research, thus exempting it from approval by the University at Buffalo Institutional Review Board.

Patient population

CPT codes related to free flap reconstruction of the head and neck were searched for use in the study [11]. Skin, fascia, muscle, and bone grafts used in head and neck surgeries were ultimately included in the study and analysis. Table 1 shows a list of the individual CPT codes used. A total of 10 CPT codes were deemed suitable in the NSQIP database, each with its surgical cases recorded.

**Table 1:** Frequently searched CPT codes CPT: Current Procedural Terminology

CPT Codes	Description of CPT code
15756	Free muscle flap with or without skin graft with microvascular anastomosis
15757	Free skin flap with microvascular anastomosis
15758	Free fascial flap with microvascular anastomosis
15840	Graft for facial nerve paralysis; free fascia graft (including obtaining fascia)
15841	Graft for facial nerve paralysis; free muscle graft (including obtaining graft)
15842	Graft for facial nerve paralysis; free muscle graft by microsurgical technique
15845	Graft for facial nerve paralysis; regional muscle transfer
15855	Bone graft with microvascular anastomosis; fibula
15856	Bone graft with microvascular anastomosis; iliac crest
20962	Bone graft with microvascular anastomosis; other than fibula, iliac crest, or metatarsal

Statistical analysis

Statistical analysis, including nearest neighbor propensity score matching, was completed using IBM SPSS Statistics for Windows, version 28 (Released 2021; IBM Corp., Armonk, New York, United States). Predictor variables utilized in this study were related to demographics and medical comorbidities. The variables utilized for outcome measurements included total hospital length of stay, reoperation events, and readmission events. Definitions of each variable are outlined in the ACS NSQIP Participant Use Data File [12].

Parametric methods were utilized for the statistical analysis. Surgical outcomes were compared by race initially. A sub-analysis was performed after propensity score matching between those who identify as being from the Caucasian (White) race and those who identify as being from the Black or African American race. Logistic regression was utilized to determine individual variable effects on reoperation and readmission events. Linear regression was used for the variable effect on length of stay. The study power was found to be 90%.

## Results

A total of 1589 patients underwent head and neck free flap surgeries between 2017 and 2019. There were 338 cases removed from the analysis when race was reported as unknown or not reported. Of the remaining 1251 patients, 1075 (85.9%) identified their race as Caucasian and 176 as non-Caucasian, of which 94 identified their race as Black or African American. Table 2 shows the demographic data of cases in this study, including sex, race, ethnicity, and age.

**Table 2:** Demographics of the patient population (N=1251)

Demographics	Frequency (Percentage)
Sex	
Male	814 (65.1)
Female	437 (34.9)
Race	
White	1075 (85.9)
Black or African American	94 (7.5)
Asian	65 (5.2)
Native Hawaiian or Pacific Islander	14 (1.1)
American Indian or Alaska Native	3 (0.2)
Ethnicity	
Hispanic	46 (3.7)
Not Hispanic	1185 (94.7)
Unknown/Not Reported	20 (1.6)
Mean Age in Years (95% CI)	60.9 (60.1-61.7)

Table 3 shows the medical and surgical comorbidities of the patients included in this study. Hypertension (n=569, 45.5%) and smoking (n=317, 25.3%) were the most notable medical comorbidities and risk factors seen in this population. However, the cohort demonstrated a substantial burden of many other comorbidities, with 951 patients (76.1%) classified as American Society of Anesthesiologists (ASA) class III or higher, reflecting a high degree of preoperative systemic disease. Open wound infections were present postoperatively in 179 patients (14.3%) in the cohort, and weight loss was also reported in 98 patients (7.8%). Postoperatively, blood transfusion was required in 292 patients (23.3%), while cardiopulmonary complications such as pneumonia were less common, presenting in just 57 patients (4.6%). Failure to wean postoperatively from mechanical ventilation occurred in 48 patients (3.8%). Table 4 shows the primary and secondary outcomes for the study, with an average length of stay of 9.49 days. A total of 107 patients (8.6%) required readmission for a related complication, and 214 patients (17.1%) required a reoperation within 30 days of the free flap reconstruction. 

**Table 3:** Comorbidities of patients in the patient population (N=1251) ASA: American Society of Anesthesiologists

Comorbidity	Frequency (Percentage)
Diabetes	
Insulin	59 (4.7)
Non-Insulin	105 (8.4)
Smoking	317 (25.3)
Dyspnea	
Moderate	74 (5.9)
At Rest	7 (0.6)
Functional Status	
Partially dependent	29 (2.3)
Fully dependent	6 (0.5)
Ventilator Dependent	4 (0.3)
COPD	72 (5.8)
Ascites	1 (0.1)
CHF	11 (0.9)
HTN w/ Med	569 (45.5)
Renal Failure	1 (0.1)
Dialysis	8 (0.6)
Disseminated Cancer	61 (4.9)
Open Wound Infection	179 (14.3)
Steroid Use	57 (4.6)
Weight Loss	98 (7.8)
Bleeding Disorder	35 (2.8)
Transfusion Required	6 (0.5)
Sepsis	
SIRS	12 (1.0)
Sepsis	5 (0.4)
ASA Class	
1	25 (1.9)
2	275 (22.0)
3	869 (69.5)
4	81 (6.5)
5	1 (0.1)

**Table 4:** Primary and secondary outcomes measured (N=1251) Data are presented as frequency (percentage) except for length of hospital stay, which is given as mean ± standard error of the mean (SEM)

Primary Outcome	Frequency (Percentage)
Length of Hospital Stay, mean ± SEM	9.49 ± 0.38 days
Reoperation	214 (17.1)
Related Readmission	107 (8.6)
Secondary Outcome	
Superficial Infection	99 (7.9)
Deep Wound Infection	34 (2.7)
Organ Space Infection	38 (3.0)
Dehiscence	62 (5.0)
Pneumonia	57 (4.6)
Unplanned Reintubation	27 (2.2)
Pulmonary Embolism	10 (0.8)
Fail to Wean from Ventilator	48 (3.8)
Renal Insufficiency	1 (0.1)
Acute Renal Failure	0
Urinary Tract Infection	9 (0.7)
Stroke/Cerebrovascular Accident	6 (0.5)
Cardiac Arrest	7 (0.6)
Myocardial infarction	10 (0.8)
Transfusion Required	292 (23.3)
Deep Vein Thrombosis	24 (1.9)
Sepsis	34 (2.7)
Septic Shock	6 (0.5)
Clostridioides difficile	13 (1.0)

Using univariate analysis, cases were initially analyzed by Caucasian and non-Caucasian patients. Following this, unmatched and matched analyses were conducted for patients who identified as White race compared to those who identified as Black or African American race. In the univariate analysis of Caucasian and non-Caucasian patients, non-Caucasian patients presented significantly younger as compared to Caucasian patients (54.7 years vs 61.7 years, respectively, p<.001). Furthermore, a higher percentage of non-Caucasian patients were ventilator-dependent compared to their Caucasian counterparts (1.1% vs 0.2%, respectively), though this difference was not statistically significant (p=.097). Interestingly, Caucasian patients had higher rates of moderate dyspnea compared to non-Caucasian patients (p=.027). Caucasian patients were functionally more dependent on others compared to non-Caucasians (p=.011). There were no significant differences found in primary or secondary outcomes in this univariate analysis (Table 5).

**Table 5:** Comparison of the Caucasian and non-Caucasian groups based on characteristics and comorbidities Data are presented as mean ± SEM or frequency (percentage). ^a ^Fisher exact test was used for analysis COPD: chronic obstructive pulmonary disease; CHF: congestive heart failure; SIRS: systemic inflammatory response syndrome; ASA: American Society of Anesthesiologists; LOS: length of hospital stay; DVT: deep vein thrombosis; HTN: hypertension

Characteristic	Unmatched (N=1251)		p-Value
	Caucasian (N=1075)	Non-Caucasian (N=176)	
Preoperative Characteristics			
Sex (male)	708 (65.9)	106 (60.2)	.146
Ethnicity (Hispanic)	43 (4.0)	3 (1.7)	.277
Age (average years)	61.7 ± 0.82	54.7 ± 2.14	< .001>
BMI (average)	26.6 ± 0.37	26.1 ± 0.90	.275
Comorbidities			
Diabetes			.093
Insulin	55 (5.1)	4 (2.3)	.099
Non-insulin	85 (7.9)	20 (11.4)	.125
Smokers	280 (26.0)	37 (21.0)	.155
Dyspnea			.049
None	1000 (93.0)	170 (96.6)	.075
Moderate	70 (6.5)	4 (2.3)	.027
At rest	5 (0.5)	2 (1.1)	.268
Functional status			.093
Independent	1040 (96.7)	176 (100)	.011
Partially dependent.	29 (2.7)	0	.027
Fully dependent	6 (0.5)	0	.320
Ventilator	2 (0.2)	2 (1.1)	.097^a^
COPD	66 (6.1)	6 (3.4)	.166
Ascites	1 (0.1)	0	1.000^a^
CHF	9 (0.8)	2 (1.1)	.660^a^
HTN with medication	490 (45.6)	79 (44.9)	.864
Renal failure	1 (0.1)	0	1.000^a^
Dialysis	6 (0.5)	2 (1.1)	.313^a^
Disseminated cancer	57 (5.3)	4 (2.3)	.090^a^
Open wound infection	154 (14.3)	25 (14.2)	.966
Steroid	52 (4.8)	5 (2.8)	.328^a^
Weight loss	86 (8.0)	12 (6.8)	.589
Bleeding disorder	34 (3.2)	1 (0.5)	.050^a^
Transfusion	5 (0.5)	1 (0.5)	.598
Sepsis			.642
SIRS	10 (0.9)	2 (1.1)	.759
Sepsis	5 (0.5)	0	.365
ASA class			.352
1	20 (1.9)	5 (2.8)	.389
2	232 (21.6)	43 (24.4)	.397
3	747 (69.5)	122 (69.3)	.964
4	75 (7.0)	6 (3.4)	.075
5	1 (0.1)	0	.686
Primary Outcome			
LOS	9.43 ± 0.42 days	9.83 ± 1.00 days	.195
Reoperation	182 (16.9)	32 (18.2)	.683
Related readmission	91 (8.5)	16 (9.1)	.904
Secondary Outcome			
Superficial infection	89 (8.3)	10 (5.7)	.237
Deep wound infection	31 (2.9)	3 (1.7)	.614^a^
Organ space infection	33 (3.1)	5 (2.8)	1.000^a^
Dehiscence	55 (5.1)	7 (4.0)	.707^a^
Pneumonia	44 (4.1)	13 (7.4)	.076^a^
Unplanned reintubation	25 (2.3)	2 (1.1)	.412^a^
Pulmonary embolism	10 (0.9)	0	.374^a^
Fail to wean from vent	39 (3.6)	9 (5.1)	.394^a^
Renal insufficiency	1 (0.1)	0	1.000^a^
Urinary tract infection	9 (0.8)	0	.623^a^
Stroke/CVA	6 (0.6)	0	1.000^a^
Cardiac arrest	7 (0.7)	0	.602^a^
Myocardial infarction	9 (0.8)	1 (0.6)	1.000^a^
Transfusion required	242 (22.5)	50 (28.4	.086
DVT	19 (1.8)	5 (2.8)	.368^a^
Sepsis	30 (2.8)	4 (2.3)	1.000^a^
Septic shock	5 (0.5)	1 (0.6)	.598^a^
Clostridioides difficile	11 (1.0)	2 (1.1)	.703^a^

Prior to propensity score matching, univariate analysis between Caucasian and Black or African American patients showed that Black or African American patients presented at a younger age than White patients (55.2 + 2.9 vs 61.7 + 0.8 years, respectively, p<.001). Black or African American patients were also more likely to have dyspnea at rest (2.1 vs 0.5%, respectively, p=.045), coinciding with Black patients being more likely to have preoperative ventilator dependence than White patients (2.1 vs 0.2%, respectively, p=.034) (Table 6). During the matching process, 88 White patients were matched to 94 Black or African American patients with the nearest neighbor propensity score matching. This process removed all significant differences in preoperative characteristics between the two groups. In both unmatched and matched univariate analysis, there was no significant difference in primary outcomes, including length of stay (LOS), reoperation, or related readmission. Additionally, there were no significant differences in post-operative complications seen between the two groups in the unmatched or matched analysis (Table 6).

**Table 6:** Comparison of preoperative characteristics, primary outcomes, and secondary outcomes between the groups Data are presented as mean ± SEM or frequency (percentage). ^a ^Fisher exact test was used for analysis COPD: chronic obstructive pulmonary disease; CHF: congestive heart failure; SIRS: systemic inflammatory response syndrome; ASA: American Society of Anesthesiologists; LOS: length of hospital stay; DVT: deep vein thrombosis; HTN: hypertension

Characteristic	Unmatched (N=1169)			Matched (N=182)		
	White (n=1075)	Black or African American (n=94)	p-value	White (n=88)	Black or African American (n=94)	p-value
Preoperative Characteristics						
Sex (male)	708 (65.9)	55 (58.5)	0.151	48 (54.5)	55 (58.5)	.590
Ethnicity (Hispanic)	43 (4.0)	2 (2.1)	0.574^a^	2 (2.3)	2 (2.1)	.529
Age (years)	61.7 ± 0.82	55.2 ± 2.91	<0.001	54.9 ± 3.41	55.2 ± 2.91	.839
BMI (kg/m^2^)	26.6 ± 0.37	27.3 ± 1.42	0.430	26.0 ± 1.37	27.3 ± 1.42	.194
Comorbidities						
Diabetes			0.485			.869
Insulin	55 (5.1)	3 (3.2)	0.410	2 (2.3)	3 (3.2)	.705
Non-insulin	85 (7.9)	10 (10.6)	0.353	11 (12.5)	10 (10.6)	.694
Smokers	280 (26.0)	26 (27.7)	0.733	26 (29.5)	26 (27.7)	.778
Dyspnea			0.096			.387
None	1000 (93.0)	88 (93.6)	0.828	84 (95.5)	88 (93.6)	.587
Moderate	70 (6.51)	4 (4.3)	0.389	4 (4.5)	4 (4.3)	.924
At rest	5 (0.5)	2 (2.1)	0.045	0	2 (2.1)	.169
Functional status			0.329			-
Independent	1040 (96.7)	94 (100)	0.064	88 (100)	94 (100)	-
Partially dependent	29 (2.7)	0	0.107	0	0	-
Fully dependent	6 (0.5)	0	0.468	0	0	-
Ventilator	2 (0.2)	2 (2.1)	0.034^a^	1 (1.1)	2 (2.1)	1.000^a^
COPD	66 (6.1)	6 (6.4)	0.825^a^	7 ((8.0)	6 (6.4)	.777^a^
Ascites	1 (0.1)	0	1.000^a^	0	0	-
CHF	9 (0.8)	1 (1.1)	0.569^a^	0	1 (1.1)	1.000^a^
HTN with medication	490 (45.6)	48 (51.1)	0.306	40 (45.5)	48 (51.1)	.449
Renal failure	1 (0.1)	0	1.000^a^	0	0	-
Dialysis	6 (0.5)	2 (2.1)	0.130^a^	0	2 (2.1)	.498^a^
Disseminated cancer	57 (5.3)	3 (3.2)	0.473^a^	2 (2.3)	3 (3.2)	1.000^a^
Open wound infection	154 (14.3)	15 (16.0)	0.666	10 (11.4)	15 (16.0)	.368
Steroid	52 (4.8)	2 (2.1)	0.309^a^	2 (2.3)	2 (2.1)	1.000^a^
Weight loss	86 (8.0)	4 (4.3)	0.230^a^	4 (4.5)	4 (4.3)	1.000^a^
Bleeding disorder	34 (3.2)	1 (1.1)	0.355^a^	1 (1.1)	1 (1.1)	1.000^a^
Transfusion	5 (0.5)	1 (1.1)	0.396^a^	0	1 (1.1)	1.000^a^
Sepsis			0.515			-
SIRS	10 (0.9)	0	0.348	0	0	-
Sepsis	5 (0.5)	0	0.508	0	0	-
ASA class			0.462			.764
1	20 (1.9)	4 (4.3)	0.116	3 (3.4)	4 (4.3)	.767
2	232 (21.6)	22 (23.4)	0.681	20 (22.7)	22 (23.4)	.914
3	747 (69.5)	64 (68.1)	0.777	58 (65.9)	64 (68.1)	.755
4	75 (7.0)	4 (4.3)	0.313	7 (8.0)	4 (4.3)	.295
5	1 (0.1)	0	0.767	0	0	-
Primary Outcome						
LOS	9.43 ± 0.42 days	9.96 ± 1.43 days	0.318	9.63 ± 1.66 days	9.96 ± 1.43 days	.453
Reoperation	182 (16.9)	16 (17.0)	0.982	10 (11.4)	16 (17.0)	.276
Related readmission	91 (8.5)	10 (10.6)	0.445^a^	8 (9.1)	10 (10.6)	.807^a^
Secondary Outcome						
Superficial infection	89 (8.3)	3 (3.1)	0.106^a^	2 (2.3)	3 (3.1)	1.000^a^
Deep wound infection	31 (2.9)	3 (3.1)	0.750^a^	1 (1.1)	3 (3.1)	.622^a^
Organ space infection	33 (3.1)	4 (4.3)	0.532^a^	2 (2.3)	4 (4.3)	.683^a^
Dehiscence	55 (5.1)	5 (5.3)	0.810^a^	6 (6.8)	5 (5.3)	.761^a^
Pneumonia	44 (4.1)	5 (5.3)	0.587^a^	3 (3.4)	5 (5.3)	.722^a^
Unplanned reintubation	25 (2.3)	1 (1.1)	0.716^a^	2 (2.3)	1 (1.1)	.611^a^
Pulmonary embolism	10 (0.9)	0	1.000^a^	1 (1.1)	0	.484^a^
Failure to wean from vent	39 (3.6)	4 (4.3)	0.772^a^	2 (2.3)	4 (4.3)	.683^a^
Renal insufficiency	1 (0.1)	0	1.000^a^	0	0	-
Urinary tract infection	9 (0.8)	0	1.000^a^	0	0	-
Stroke/Cerebrovascular accident	6 (0.6)	0	1.000^a^	0	0	-
Cardiac arrest	7 (0.7)	0	1.000^a^	1 (1.1)	0	.484^a^
Myocardial infarction	9 (0.8)	1 (1.1)	0.569^a^	1 (1.1)	1 (1.1)	1.000^a^
Transfusion required	242 (22.5)	22 (23.4)	0.843	23 (26.1)	22 (23.4)	.669
DVT	19 (1.8)	3 (3.1)	0.413^a^	0	3 (3.1)	.247^a^
Sepsis	30 (2.8)	2 (2.1)	1.000^a^	4 (4.5)	2 (2.1)	.423^a^
Septic shock	5 (0.5)	0	1.000^a^	0	0	-
Clostridioides difficile	11 (1.0)	1 (1.1)	1.000^a^	2 (2.3)	1 (1.1)	.611^a^

Logistic regression for reoperation and readmission, and linear regression for LOS were completed in a multivariate analysis. All variables in Tables 2, 3 were included, as well as patient BMI data. Logistic regression for reoperation showed no variables with a significant impact on reoperation events. Males were more likely to be readmitted compared to females (OR=3.9, 95% CI=1.0-14.9, p=.046). Linear regression showed male sex (β=2.3, 95% CI=0.1-4.5, p=.037), chronic obstructive pulmonary disease (COPD) (β=4.8, 95% CI=0.5-9.1, p=.029), and infection (β=4.8, 95% CI=1.5-8.0, p=.004) were associated with increased LOS, while patients taking medication for hypertension had a decreased LOS (β=-3.6, 95% CI=-6.0 to -1.1, p=.004). Significant results of regression analysis are presented in Table 7.

**Table 7:** Regression of readmission and length of stay COPD: chronic obstructive pulmonary disease; HTN: hypertension; LOS: length of stay

Regression Type	Odds Ratio	95% CI	p-value
Readmission			
Male	3.9	1.0 to 14.9	.046
LOS			
Risk Factor	Increase in LOS (days)	95% CI	p-value
Male	2.3	0.1 to 4.5	.037
COPD	4.8	0.5 to 9.1	.029
HTN with Medication	-3.6	-6.0 to -1.1	.004
Open Wound Infection	4.8	1.5 to 8.0	.004

## Discussion

Relatively few complications were reported overall, with many postoperative complications occurring in less than 1% of operations, which demonstrates the high success rates of free flap reconstructions that were previously reported [3]. Most complications were related to bleeding/transfusion, postoperative infections, and wound dehiscence. Wound infection rates have varied between studies, having been reported as low as 8% to as high as 41% [3]. The infection rates in this study were on the lower end of this spectrum, with 99 (7.9%), 34 (2.7%), and 38 (3.0%) operations having superficial incisional, deep incisional, or organ space infections, respectively. Current recommendations in the American Society of Health-System Pharmacists guidelines suggest using clindamycin for patients undergoing clean-contaminated head and neck surgeries who have β-lactam antibiotic allergies [13]. In one study assessing various antibiotic regimens in free flap reconstructions, clindamycin was shown to be associated with higher rates of infection when used alone compared to the use of other combination antibiotic regimens [13]. NSQIP does not report on the antibiotics used in each case, preventing analysis of specific antibiotic use and respective infection rates in our study. 

Readmissions were most often related to wound infections, whereas reoperations were often related to vascular disruption to the flap or infection [4]. In this study, the level of postoperative care was not reported, but the high rates of reoperation related to vascular revision indicate the importance of frequent flap checks and early management of vascular compromise. Many preoperative comorbidities, such as tobacco use, diabetes, and cardiovascular disease, have been implicated in poor surgical outcomes for free flap reconstruction. Abouyared et al., in particular, determined that malnutrition, tobacco use, and diabetes were associated with higher rates of failure and infections in patients undergoing free flap reconstruction [4]. In our study, COPD and preoperative wound infection were shown to increase LOS but were not associated with increases in 30-day readmission or reoperation. Patients taking medication for hypertension were shown to have a shorter hospital stay. This correlates with Wang et al., who described the positive relationship between transiently elevated systolic blood pressure and the risk of hematoma formation [14]. Consequently, proper management of hypertension can contribute to a reduced LOS.

Studies have indicated that the incidence of head and neck cancer by race has changed over time. In the late 1990s and early 2000s, African American populations were found to have a 47-65% higher incidence of head and neck cancer as compared to White populations [15]. In addition to the difference in incidence, African American patients were more likely to present at an earlier age, have more advanced disease on presentation, and have metastatic disease [15]. These outcomes are consistent with our study, with Black patients presenting significantly younger than White patients, albeit disease staging was not recorded in our data analysis. The absence of disease staging within our analysis could be the reason there are significantly fewer Black patients in the database who have undergone free flap reconstruction, but these conclusions cannot be drawn from the data in this study.

More recent studies have shown changes in the incidence of head and neck cancers based on sex and race. Since 2009, the incidence of head and neck cancers in African American individuals has been decreasing at a faster rate than in White American individuals, as evidenced by fewer diagnoses of head and neck squamous cell carcinoma in Black women with non-oropharyngeal head and neck squamous cell carcinoma [16]. In addition, there is a convergence in the frequency of diagnoses of oropharyngeal and non-oropharyngeal head and neck cancers between Black and White men [16]. While our study did not directly compare racial incidences of head and neck cancer or free flap surgery, there was a significantly higher proportion of White patients undergoing free flap reconstruction in the database used. This appears to be a common limitation in studies comparing race in head and neck cancer, as other studies have attempted to limit this discrepancy [15].

Black or African American patients tend to have greater mortality from cancer, including head and neck cancer. In 2000, African American men had an 85% higher mortality rate from oral cancer as compared to their White counterparts [17]. This discrepancy could be attributed to several factors, including genetics, delayed presentation, barriers to care, and differences in quality of care [17]. When comparing disease-specific five-year survival between Black and White American patients, access to healthcare and insurance were found to be significant contributing factors to increased mortality among Black American patients [15]. Due to the increase in mortality, it is important to determine if there are racial differences in postoperative outcomes when matching to control for preoperative variability. In our study, there were no differences found in primary outcomes based on racial differences between White and Black or African American patients. While this study did not directly investigate racial differences in pre-diagnosis or preoperative characteristics, it is of note that White patients had their operation at a significantly older mean age than patients of other races. Further research will be required to draw conclusions on this discrepancy. Based on our results, however, it does not appear that race is associated with differences in early morbidity after free flap reconstruction.

Limitations

NSQIP only collects data 30-days post operation, so significant morbidity and mortality related to surgical outcome may have occurred following this date. Due to the nature of head and neck cancer and surgical reconstruction, vital functions and quality of life that would not be reported in 30-day outcomes may be significantly underreported. With the high rate of success of free flaps, complications are limited. This could lead to difficulty accurately analyzing complications, leading to false negatives that would not be realized with small populations for each individual complication.

Missing variables made some analyses not realistic for this study. It has been reported that malnutrition has played a significant role in postoperative outcomes, specifically in head and neck cancer [13]. The location of the cancer and reconstruction can often make swallowing difficult, leading to significant malnutrition perioperatively. Unfortunately, preoperative and postoperative malnutrition data were scarce for most of the analyzed cases. While albumin, prealbumin, and C-reactive protein levels could be used to help measure malnutrition, most of the records did not contain any reported blood work or lab values. 

As previously stated, postoperative level of care and antibiotics have a significant impact on postoperative outcomes in these patients. Frequent flap checks are required to adequately detect vascular compromise early. Antibiotics also have an important role in free flap reconstruction, as postoperative infection is a common cause of reoperation and readmission. Unfortunately, neither of these variables is collected in NSQIP. Additionally, there are many factors that go into the decision-making of free flap reconstruction, including donor site, cancer site, and characteristics, and doctor-patient shared decision-making, that are absent from this database. 

Most of the significant risk factors for head and neck cancer are not reported in NSQIP, including HPV and EBV status. While tobacco use was not found to have a significant impact on postoperative outcomes in this study, looking at the other previously described risk factors could help to determine which, if any, influence postoperative outcomes.

## Conclusions

In patients included in the NSQIP database, race does not appear to impact outcomes of patients undergoing free flap reconstruction of the head and neck. This is what we should expect in local case series as well. NSQIP is an imperfect dataset, so any conclusions drawn need to be confirmed with other methodologies. 
